# Interpretable Machine Learning Prediction of Drug-Induced QT Prolongation: Electronic Health Record Analysis

**DOI:** 10.2196/42163

**Published:** 2022-12-01

**Authors:** Steven T Simon, Katy E Trinkley, Daniel C Malone, Michael Aaron Rosenberg

**Affiliations:** 1 Division of Cardiology University of Colorado School of Medicine Aurora, CO United States; 2 Department of Clinical Pharmacy School of Pharmacy University of Colorado Aurora, CO United States; 3 College of Pharmacy University of Utah Salt Lake City, UT United States; 4 Division of Cardiac Electrophysiology University of Colorado School of Medicine Aurora, CO United States

**Keywords:** drug-induced QT prolongation, predictive modeling, interpretable machine learning, ML, artificial intelligence, AI, electronic health records, EHR, prediction, risk, monitoring, deep learning

## Abstract

**Background:**

Drug-induced long-QT syndrome (diLQTS) is a major concern among patients who are hospitalized, for whom prediction models capable of identifying individualized risk could be useful to guide monitoring. We have previously demonstrated the feasibility of machine learning to predict the risk of diLQTS, in which deep learning models provided superior accuracy for risk prediction, although these models were limited by a lack of interpretability.

**Objective:**

In this investigation, we sought to examine the potential trade-off between interpretability and predictive accuracy with the use of more complex models to identify patients at risk for diLQTS. We planned to compare a deep learning algorithm to predict diLQTS with a more interpretable algorithm based on cluster analysis that would allow medication- and subpopulation-specific evaluation of risk.

**Methods:**

We examined the risk of diLQTS among 35,639 inpatients treated between 2003 and 2018 with at least 1 of 39 medications associated with risk of diLQTS and who had an electrocardiogram in the system performed within 24 hours of medication administration. Predictors included over 22,000 diagnoses and medications at the time of medication administration, with cases of diLQTS defined as a corrected QT interval over 500 milliseconds after treatment with a culprit medication. The interpretable model was developed using cluster analysis (K=4 clusters), and risk was assessed for specific medications and classes of medications. The deep learning model was created using all predictors within a 6-layer neural network, based on previously identified hyperparameters.

**Results:**

Among the medications, we found that class III antiarrhythmic medications were associated with increased risk across all clusters, and that in patients who are noncritically ill without cardiovascular disease, propofol was associated with increased risk, whereas ondansetron was associated with decreased risk. Compared with deep learning, the interpretable approach was less accurate (area under the receiver operating characteristic curve: 0.65 vs 0.78), with comparable calibration.

**Conclusions:**

In summary, we found that an interpretable modeling approach was less accurate, but more clinically applicable, than deep learning for the prediction of diLQTS. Future investigations should consider this trade-off in the development of methods for clinical prediction.

## Introduction

Drug-induced long-QT syndrome (diLQTS) [1,2] is a major concern for inpatients worldwide and has been identified as a key target for clinical decision support tools [3-7]. Importantly, although certain medications have been implicated as having significant clinical risk [8,9], for others, despite a known risk of diLQTS, clinical validation has been lacking [10-12]. In the past few years, several groups have sought to apply prediction models using electronic health record (EHR) data to model risk [13-17] toward the goal of developing an automated approach that leverages innovations in data science and machine learning. In prior work [18], we performed a comparative evaluation of machine learning methods to predict diLQTS using EHR data, in which we found that the most accurate prediction method was a deep learning model (6-layer neural network). However, each of the models carried the limitation of lacking interpretability for its predictions [19], as we were unable to assess which clinical features were the most predictive. As such, we were unable to construct a meaningful decision support approach based on these models to reduce the risk of diLQTS or determine whether our model could be easily exported to other systems.

Beyond the role of increasing trust [20] in a prediction model, interpretability plays a critical role in the assessment of prediction models [21], particularly in the age of artificial intelligence, where increasingly complex models can be created using relatively raw, or unprocessed, clinical features. Limitations in interpretability are critical not only because the users may not understand why a model makes the recommendations that it does but also because a lack of interpretability increases the risk of bias in the form of data shifts [22-24]. Data shifts occur when a model is developed in one population and then applied in a different population; note that this effect could also occur within the same hospital system if the treatment paradigm changes dynamically over time. The inclusion of interpretable models also allows a detailed investigation to uncover confounding and identify situations where a critical factor was excluded from the prediction framework and to assess for reverse causality, a critical consideration in big data models. Although “interpretability” itself cannot be well quantified in the same manner as accuracy or calibration, it remains a critical consideration in the development of predictive models.

The promise of EHR data is that it provides a scale (ie, power) to draw clinical inferences across thousands of patients and potentially millions of data points, at the cost of lacking the ability for facile clinical validation. With this power comes the ability to predict clinical outcomes across a large number of heterogenous subjects, integrating the breadth of the clinical record and, with it, the range of possible diagnoses and medications that could have nonlinear associations that cannot be as easily detected using standard (ie, regression-based) methods. However, methods to leverage EHR data using machine learning have been limited by the ability to include interpretability along with predictive accuracy.

In this follow-up investigation to our previous work [18], we examined the application of an interpretable approach to predictive modeling applied at scale to EHR data to predict diLQTS. We specifically examined the use of clustering as a bridge to interpretability and compared this approach with a deep learning, noninterpretable method previously identified as providing superior predictive accuracy within our health care system.

## Methods

### Data Source and Study Population

**Figure 1:**
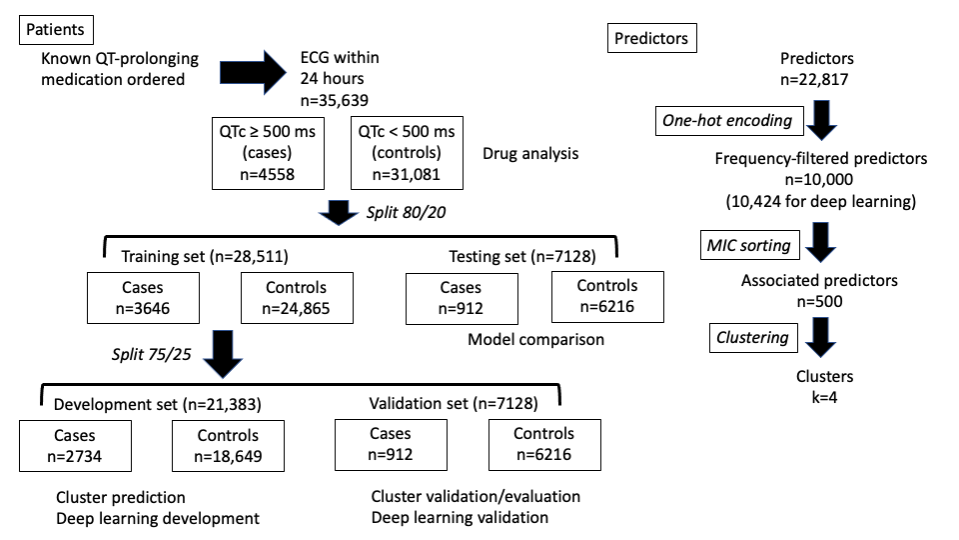
Data management schema. Left: patient data ascertained by order for known QT-prolonging medication with an electrocardiogram (ECG) performed within 24 hours to define cases (QTc ≥500 ms) and controls (QTc <500 ms), followed by subsequent splitting for models and validation. All splits stratified by case status. Right: processing of predictors using frequency filters, information coefficient, and clustering. MIC: maximum information coefficient; QTc: corrected QT interval.

The data for this investigation have been previously described [19]. Briefly, we examined EHR data from 35,639 inpatients within the UCHealth system treated between 2003 and 2018 with at least 1 of 39 medications associated with the risk of drug-induced QT prolongation and who had an electrocardiogram (ECG) in the system performed within 24 hours of medication administration (Figure 1). The primary outcome of drug-induced QT prolongation was based on any corrected QT interval over 500 milliseconds during the encounter, after the exclusion of ECGs with conduction disease (eg, bundle branch block, intraventricular conduction disease, and ventricular pacing). Predictors included any medication or diagnosis (International Classification of Diseases, Ninth or Tenth Edition) listed in the medical record that was present at the time of medication administration.

### Initial Drug Analysis

Varying formulations for each of the 39 culprit medications were combined (ie, oral and intravenous amiodarone were analyzed together). We first performed an unadjusted association analysis with each medication and the risk of diLQTS using a chi-square calculation. Those with significant associations after adjustment for multiple comparison (Bonferroni correction, *P* value for significance = .05/29 = .0017) were categorized as “high risk” for a combined analysis, as well as further model development (see below).

### Predictor Filtering and Data Splitting

The medications and diagnoses in the raw data set were extracted from the EHR for each subject as a string array, following which we performed one-hot encoding (*keras.Tokenizer* [25]; version 2.8.0) to create a separate variable for each, labeled as 0 if the diagnosis or medication was absent at the time of QT-associated medication administration and 1 if it was present. As such, missing values were coded as 0, under the assumption that if the medication or diagnosis was not present in the EHR, the patient was not taking the medication or did not have that diagnosis. This process resulted in a data set containing 22,817 unique medications and diagnostic codes, from which we filtered the top 10,000 based on frequency. Of note, the 10,000th most frequent predictor was present in only 5 of 36,639 subjects. The unadjusted association for each of these 10,000 predictors with diLQTS was examined using the maximum information coefficient (MIC; *minepy.MIC*; version 1.2.6), which examines both linear and nonlinear associations based on mutual information [26]. After sorting by MIC, the top 500 most associated diagnoses and medications were selected for cluster analysis (see below). For deep learning analysis, the top 10,424 predictors after one-hot encoding were directly inputted into the model. Data splitting (Figure 1) was performed by subject index, stratified by the diagnosis of diLQTS (*sklearn.train_test_split*; version 1.1.2). The data were first split into training (28,511/35,639, 80%) and testing (7128/35,639, 20%) sets; the training set was then further split into development (21,383/28,511, 75%) and validation (7128/28,511, 25%) sets. The development set was used to fit clusters (cluster analysis) as well as to train the deep neural network. The validation set was used to examine cluster patterns and predictive accuracy, as well as to examine the training of deep learning. The testing set was used for comparative testing of cluster and deep learning models as outlined below.

### Cluster Development and Evaluation

Clustering was performed using only diagnostic codes to facilitate comparisons of risk by drugs. To identify the optimal number of clusters, we first applied KMean clustering (*sklearn*; version 1.1.2) to the development set to create clusters from K=2 to K=50 and then examined inertia plot and silhouette scores (Figures S1A and S1B in Multimedia Appendix 1). After identification of K=4 as the optimal cluster number, we fitted the validation set with cluster assignments. To identify which diagnoses were the most overrepresented in each cluster (ie, which were the most different from other clusters), we calculated the proportion of each diagnosis for each cluster and assigned a value based on the product of the proportion within that cluster and the difference between this proportion and the cluster with the next highest proportion (termed the “proportion product”). The clinical interpretation of each cluster was performed by a clinician expert (MAR) after ranking the proportion product within each cluster. Clinical interpretation included evaluating each cluster for themes of diagnoses (eg, critical care–related diagnoses and gastrointestinal-related diagnoses) to provide an overarching framework of the “types” of patients that each cluster was composed of. Clusters were examined using chi-square test for independent association the risk of diLQTS, as well as using logistic regression (unpenalized) for the proportionate risk of any high-risk medication or combinations of high-risk medications. Margin plots were created using Stata IC software (version 16; StataCorp).

### Deep Learning Model Development

Hyperparameters for the deep learning model (deep neural network) were applied from our prior investigation [19]. Specifically, the deep neural network was composed of 6 layers, with 1024 neurons in the first layer and 512 neurons in the subsequent 5 layers; sigmoid activation function; 50% dropout for each layer; and batch normalization between layers. The final output was a binary prediction (the presence of diLQTS), with a binary cross-entropy loss function (*RMSprop optimizer*; learning rate=1 × 10^-5^; ρ=0.9), and a validation metric of area under the receiver operating characteristic curve (AUC). The model was run over 500 planned epochs, with early stopping (*keras.callbacks.EarlyStopping*) if no improvement over 50 epochs, resulting in 118 total epochs of training. Training was monitored using learning curves (Figures S2A and S2B in Multimedia Appendix 1). The development set was used for training, and the validation set was used for validation after each epoch. In total, the deep learning model had 12,265,473 total parameters, with 12,258,305 trainable parameters and 7168 nontrainable parameters.

### Model Comparison

Prediction from the cluster model was performed on the held-out testing set using logistic regression by cluster and the number of high-risk medications to obtain a predicted probability. Prediction from the deep learning model was performed through the application of the trained model to the testing set to obtain a predicted probability of diLQTS. Models were first compared using AUC, average precision score (*sklearn.metrics.average_precision_score*), and area under precision recall curve to obtain a threshold-independent comparison. The optimal probability cutoff was selected for each using the method of Youden [27]. After the selection of a cutoff, models were then compared on classification accuracy using *F*_1_-score, recall, precision, and contingency tables. Calibration was assessed using calibration curves. Platt rescaling was performed on neural network predictions through the creation of a logistic regression model to predict actual labels.

### Analysis

All analyses were conducted using Python (version 3.9.7; Python Software Foundation), run on Jupyter Notebook (Anaconda). Graphs for margin plots for cluster analysis and rescaling was performed using Stata IC software (version 16). The final script is available in Table S1 in Multimedia Appendix 1.

### Ethics Approval

This project was approved by the University of Colorado Internal Review Board (COMIRB #18-0251).

## Results

### Initial Drug Analysis

In the initial medication evaluation, we found that amiodarone, dofetilide, fluconazole, propofol, and sotalol were significantly associated with unadjusted increased risk for diLQTS (Table 1 and Table S1 in Multimedia Appendix 1). Interestingly, medications previously highly associated with inpatient diLQTS, such as haldoperidol [5], methadone [8], citalopram [28], and azithromycin [29], were either borderline or not significantly associated with diLQTS. Additionally, it was noteworthy that ondansetron [30] was significantly associated with a decreased risk of diLQTS (*P*=1.12 × 10^-39^).

**Table 1:** Association with drug-induced long-QT syndrome for selected medications. Statistically significant associations emphasized with italics.

QT-associated medication	Odds ratio (95% CI)	Chi-square (*df*)	*P* value
*Dofetilide*	*5.75 (4.68-7.06)*	*354.80 (4)*	*1.61 × 10* ^-75^
*Amiodarone*	*4.41 (4.0-4.87)*	*1010.70 (4)*	*1.69 × 10* ^-217^
*Sotalol*	*2.88 (2.28-3.65)*	*85.04 (4)*	*1.49 × 10* ^-17^
*Propofol*	*2.71 (2.49-2.96)*	*541.36 (4)*	*7.58 × 10* ^-116^
*Fluconazole*	*1.39 (1.21-1.59)*	*22.25 (4)*	*1.78 × 10* ^-4^
Methadone	1.39 (1.10-1.76)	7.45 (4)	.11
Citalopram	1.19 (1.00-1.40)	4.46 (4)	.35
Haloperidol	1.10 (1.00-1.21)	3.54 (4)	.47
Azithromycin	0.99 (0.88*-*1.12)	0.0085 (4)	.99
*Ondansetron*	*0.65 (0.61-0.69)*	*188.49 (4)*	*1.12 × 10* ^-39^

### Association With diLQTS

Among the top 10,000 most common diagnoses and medications, the 100 with the highest MIC for association with the label of diLQTS are listed in Table S2 in Multimedia Appendix 1, with the top 500 kept for cluster analysis (minimum MIC 0.000443). The top diagnoses associated with diLQTS included long-QT syndrome, acidosis, cardiogenic shock, atrial fibrillation, and acute respiratory failure; the top medications associated included potassium chloride, furosemide, amiodarone, magnesium, and albumin (Table S2 in Multimedia Appendix 1). These results highlight the potential for possible reverse causation, as it seems more likely that potassium chloride and magnesium would be administered as treatment of or to prevent diLQTS, rather than themselves being causative. The strong association with a prior diagnosis of long-QT syndrome provides a meaningful proof of principle, as congenital long-QT syndrome is a well-known risk factor for diLQTS [1,31-34].

### Cluster Analysis

**Figure 2:**
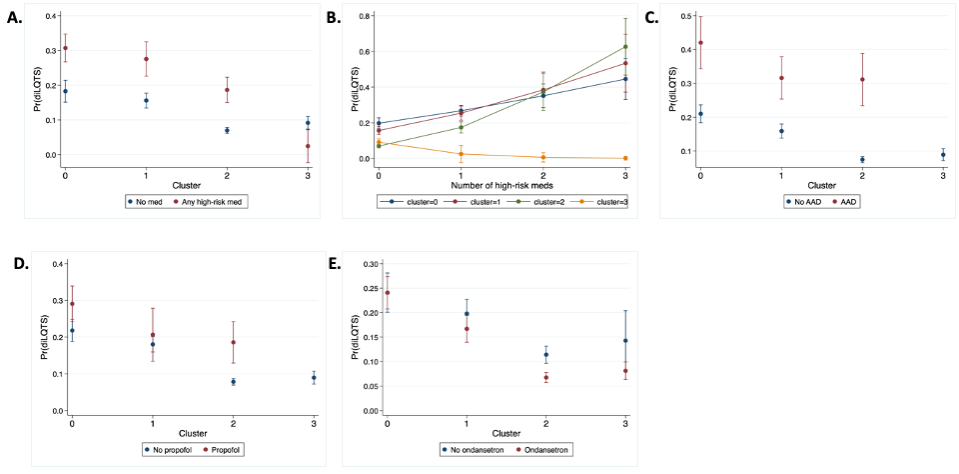
Probability of diLQTS. (A) Probability of diLQTS for each cluster with treatment with high-risk medication. (B) Probability of diLQTS with increasing numbers of high-risk meds, by cluster. (C) Probability of diLQTS for each cluster with treatment with antiarrhythmic medication (AAD). (D) Probability of diLQTS for each cluster with treatment with propofol. (E) Probability of diLQTS for each cluster with treatment with ondansetron. diLQTS: drug-induced long-QT syndrome.

Cluster number optimization identified 4 clusters as the highest silhouette score (Figure S1A in Multimedia Appendix 1), which was validated using the elbow method applied to the inertia score (Figure S1B in Multimedia Appendix 1). Manual inspection of the cluster components (Table 2 and Table S3 in Multimedia Appendix 1) indicated that cluster 0 seemed to include a large number of critical care diagnoses; cluster 1 included diagnoses suggestive of cardiovascular disease; cluster 2 included diagnoses consistent with drug intoxication and injuries; and cluster 3 included diagnoses of nausea, abdominal pain, and headaches. In the validation set, we found that clusters 0 and 1 had an increased baseline risk of diLQTS compared with clusters 2 and 3 (Table 2), which increased with exposure to high-risk medications (Figure 2A) and combinations of high-risk medications (Figure 2B). Subjects in cluster 3 were not treated with any of the high-risk antiarrhythmic medications (amiodarone, sotalol, or dofetilide), but for all 3 other clusters, treatment with one of these agents increased the risk of diLQTS (Figure 2C). Interestingly, the use of propofol was only significantly (*P*=.0002) associated with risk of diLQTS for subjects in cluster 2 (Figure 2D) but not clusters 0 (*P*=.0161) or 1 (*P*=.4920; cluster 3 was not exposed), and the use of ondansetron was significantly associated with decreased risk of diLQTS in cluster 2 (*P*=6.371 × 10^-6^) but not the other clusters (0: *P*=.996, 1: *P*=.129, and 3: *P*=.0577; Figure 2E). These results indicate that although antiarrhythmic drugs increased the risk of diLQTS broadly across all clusters, for non-antiarrhythmic medications, the impact was primarily seen in cluster 2, where propofol increased the risk of diLQTS and ondansetron decreased risk.

**Table 2:** Cluster composition and association with drug-induced long-QT syndrome (diLQTS). Cluster 3 represents baseline comparator group (odds ratio for the risk of diLQTS are compared with cluster 3).

Cluster	Representative diagnoses	Odds ratio (95% CI)	*P* value
0	Kidney failure, sepsis, respiratory failure, and anemia	3.25 (2.51-4.21)	<.001
1	Coronary artery disease, hypertension, hyperlipidemia, diabetes, and myocardial infarction	2.29 (1.77-2.95)	<.001
2	Live birth, motor vehicle accident, drug overdose, and alcohol intoxication	0.94 (0.73-1.20)	.61
3	Nausea, abdominal pain, and headache	1	N/A^a^

^a^N/A: not applicable.

### Comparison of Predictive Accuracy

**Figure 3:**
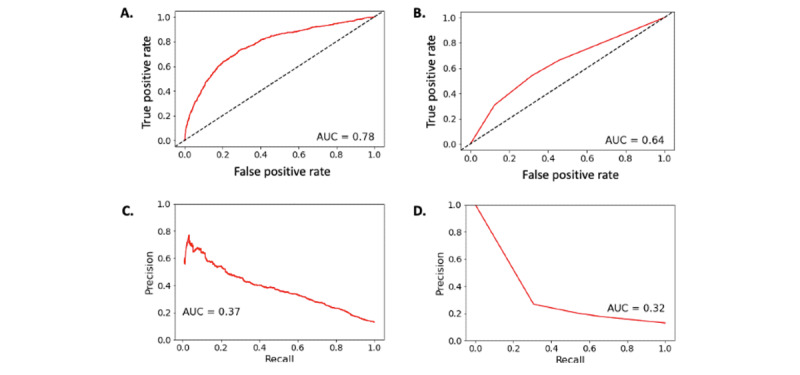
Accuracy assessment of models. (A) receiver operating characteristic (ROC) curve for neural network. (B) ROC curve for cluster model. (C) Precision-recall for neural network. (D) Precision-recall for cluster model. AUC: area under ROC curve; NN: neural network.

**Figure 4:**
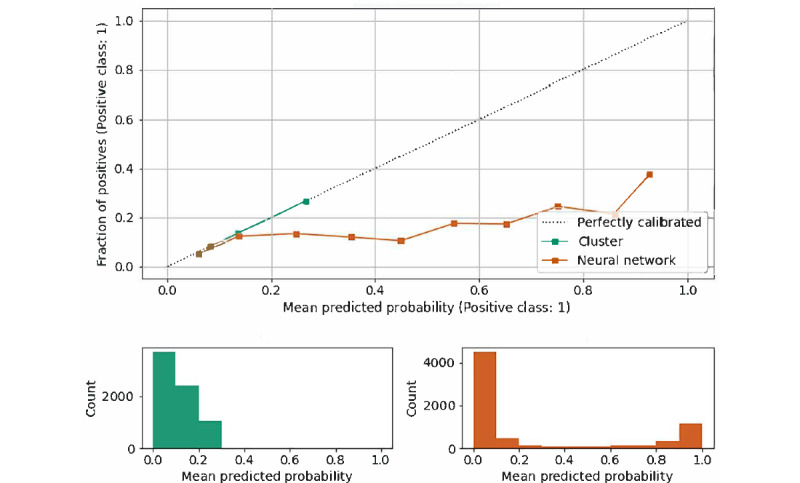
Calibration analysis of neural network and cluster-based models. Top: Calibration plot for each model, with abscissa corresponding to the binned predicted probability of diLQTS (positive class) from the model and ordinate corresponding to the proportion of actual positives (diLQTS cases) within each bin. Bottom: Histogram of predicted probability for each model (left: cluster, right: neural network). Note that cluster-based model did not predict probability over 0.5 for any individual. diLQTS: drug-induced long-QT syndrome.

The AUC for deep learning was 0.776 (Figure 3A) compared with the AUC of the cluster analysis of 0.636 (Figure 3B); the area under precision recall curve was 0.373 for deep learning (Figure 3C) compared with 0.322 for cluster analysis (Figure 3D); and the average precision score for deep learning was 0.379 and 0.193 for cluster analysis. Based on the Youden’s method for cutoff selection, the optimal cutoff for the prediction of diLQTS from deep learning was Pr(diLQTS) of 0.12, and for cluster analysis, it was 0.15. Based on these cutoffs, the *F*_1_-score for deep learning was 0.39, and for cluster analysis, it was 0.29. Contingency tables for both are in Tables S4A and S4B in Multimedia Appendix 1, with classification comparison in Table 3 demonstrating an agreement of 71.4% for the 2 approaches. Calibration comparison is provided in Figure 4, in which we noted that the neural network was poorly calibrated and generally overpredicted the risk of diLQTS (ie, actual proportion of diLQTS cases less than predicted probability), which had been described with these models in our previous work [18]. With Platt rescaling (Figures S3A and S3B in Multimedia Appendix 1), calibration of the neural network was improved and was similar to calibration of the cluster analysis (Figure S3B in Multimedia Appendix 1).

**Table 3:** A 2 × 2 table of comparative predictions at selected cutoffs. For deep learning models, the cutoff was probability of drug-induced long-QT syndrome (diLQTS) of 0.12, and for cluster analysis, it was 0.15. These values are based on predictive models for which the probability of diLQTS is produced for each individual, and the cutoff represents the probability above, in which an individual would be predicted to be at risk, and below, in which one would not be at risk.

		Cluster model (N=7128)		
		Predicted low risk, n (%)	Predicted high risk, n (%)	Total, n (%)
**Neural network model**				
	Predicted low risk	3653 (51.2)	1018 (14.3)	4671 (65.6)
	Predicted high risk	1017 (14.3)	1440 (20.2)	2457 (34.4)
	Total	4670 (65.5)	2458 (34.5)	7128 (100)

## Discussion

### Principal Findings

In this EHR-based follow-up analysis, we sought to compare 2 divergent methods for the integration of machine learning to guide clinical decisions to prevent diLQTS, with a focus on clinical interpretability and predictive accuracy. In one, we applied cluster analysis to group individuals by patterns of diagnostic codes to identify potentially recognizable clinical subgroups from which a treating clinician could identify patients who might be at risk for diLQTS to guide future decision-making. For comparison, we applied a deep learning algorithm that was identified based on prior work in this same population to obtain a “gold standard” level of predictive accuracy, to quantify the potential loss in predictive accuracy with the use of a more interpretable methodology. From a clinical perspective, our findings revealed some interesting insights regarding which specific medications have the greatest risk of diLQTS, as well as which subpopulations appear to be the most susceptible. However, we also found that there was a fairly substantial loss of predictive accuracy using this interpretable method in comparison with a “black box” method, which should be considered in future work on the integration of predictive models in clinical care.

Among the clinical insights, several are noteworthy. First, we found that when examined independent of patient characteristics, certain medications such as haldoperidol or methadone, which are well established with diLQTS, were not associated with increased risk, whereas others, such as ondansetron, were actually associated with decreased risk in our population. This finding points to the multifactorial nature of diLQTS, highlighting the need to consider other relevant contextual factors in assessing risk. However, it may also suggest that in the inpatient setting, there might be more benefit than risk with using these medications, which is also consistent with prior studies [9-12], including one where a clinical decision support tool to prevent diLQTS had a paradoxical decrease in mortality for patients in whom the treating provider ignored the alert and prescribed the known QT-prolonging medication despite risk [4]. Particularly in subjects who were not critically ill (not in cluster 0) and without a history of cardiovascular disease (not in cluster 1), there appeared to be more benefit to using ondansetron, balanced against more risk with using propofol. However, these insights should be taken with caution, as we do not know the specific timing of the administration of QT-associated medications in relation to obtaining the ECG nor whether a medication was administered once, several times, or not at all (merely listed as an as needed pro re nata medication). Such a limitation seems likely for several of the known QT-associated medications that are frequently ordered pro re nata, such as haldoperidol and ondansetron, in which we found no (former) or an inverse (latter) association with QT-prolongation. Regardless of the underlying impact, this consideration highlights the limitations of the use of clinical decision support tools applied broadly across all medications associated with diLQTS and a need to focus on the relative population risk and indication when designing future tools.

Second, we found that, perhaps not surprisingly, the cluster of patients (cluster 0) with diagnoses suggestive of critical illness were the most susceptible to use of high-risk medications for diLQTS, and that patients in clusters 2 and 3 with more benign diagnoses were less likely to have diLQTS. This finding has direct clinical implications, as it suggests that decision support tools might be the most effectively targeted toward patients in an intensive care unit, where risk is the greatest, rather than broadly across all inpatients, with the caveat that the use of propofol might need to be more closely monitored in subjects without cardiovascular disease or critical illness. Our findings also suggest that specific combinations of medications, such as amiodarone and propofol, should either be avoided or administered with close monitoring and aggressive treatment of other factors that could predispose risk of diLQTS, such as electrolyte abnormalities.

Finally, our findings highlight the critical trade-off between model interpretability and accuracy, as we found that a black-box prediction model using deep learning was significantly more accurate (greater AUC and area under precision recall curve) than the more interpretable cluster-based model. This finding raises a key question for all practitioners of predictive modeling: Is the improvement in predictive accuracy worth the lack of understanding for why the model makes the predictions it does? More specifically, without understanding how a model makes its predictions, how can it be challenged if a treating clinician believes it is less applicable for a particular patient, and what changes should be made if the predictive accuracy diminishes (a so-called “data shift” occurs [23,24]). It is not difficult for an experienced clinician to understand why patients who are critically ill (cluster 0) would be at increased risk or why combinations of medications with high risk of diLQTS would increase risk, and a method that can uncover these categories would seem to be more useful clinically than a black-box approach. Such clinical interpretation is unavailable for the deep learning model, which creates a challenge of trust in application. Further, in prior work, we demonstrated that reinforcement learning can be applied to cluster-based decision models (using a Q table) to allow a decision support tool to improve over time [35]; it is unclear whether a deep learning model could be as easily integrated with reinforcement learning or whether there would be sufficient prospective data to update the over 20 million parameters of such a model. Broadly, as increasing numbers of predictive models based on deep learning are applied to predict diLQTS, especially those applied directly to the ECG tracing itself [36,37], the trade-off with interpretability will remain a critical consideration in clinical applications.

### Limitations

Principal among the limitations of this investigation is the high degree of noise inherent in studies of EHR data at scale and the challenges with having a lack of ability to perform detailed validation of diagnoses, medications, or outcomes, beyond what can be performed in silico without manual chart review. Several of these limitations related to reverse causation or lack of temporal granularity with medication administration are highlighted above. On the one hand, this common limitation of big data science limits what can be done in terms of granular validation; on the other hand, it provides both the improvement in statistical power for modeling and some protection against population bias, as might occur with studies at a single clinic or single provider level. With the increased expansion of EHR use worldwide, it is likely that methods to explore interpretability within these large data models will be increasingly relevant, for which our investigation should provide some foundation for how interpretability can be balanced against predictive accuracy.

### Future Directions

Importantly, our findings provide the opportunity for direct clinical implementation of “smart” clinical decision tools that incorporate patient characteristics along with an understanding of patient risk to improve the accuracy of predictions of diLQTS, as well as guide clinical decisions including monitoring for those at high risk or selecting alternative agents where they are available. When combined with dynamic learning models, such as Q learning [35], our approach offers the opportunity to improve overall patient safety and clinical outcomes.

### Conclusion

In summary, we found that interpretable methods to predict diLQTS allow for evaluation in a manner that facilitates deeper inspection of specific medication interactions and the identification of meaningful clinical populations to target for prevention. This interpretability comes at the expense of predictive accuracy, which must be considered among organizations seeking to integrate predictive modeling into clinical decision support tools to prevent diLQTS.

## Appendix

Multimedia Appendix 1 Supplemental Documents.

## Abbreviations

AUC: area under the receiver operating characteristic curve
diLQTS: Drug-induced long-QT syndrome
ECG: electrocardiogram
EHR: electronic health record
MIC: maximum information coefficient
