# Supplementary material for: Interpretable Machine Learning Prediction of Drug-Induced QT Prolongation: Electronic Health Record Analysis
Source: J Med Internet Res. 2022 Dec 1;24(12):e42163. doi: 10.2196/42163 (PMC9756119; doi:10.2196/42163)
Supplement: Multimedia Appendix 1 [file jmir_v24i12e42163_app1.pdf]

Figure S1A

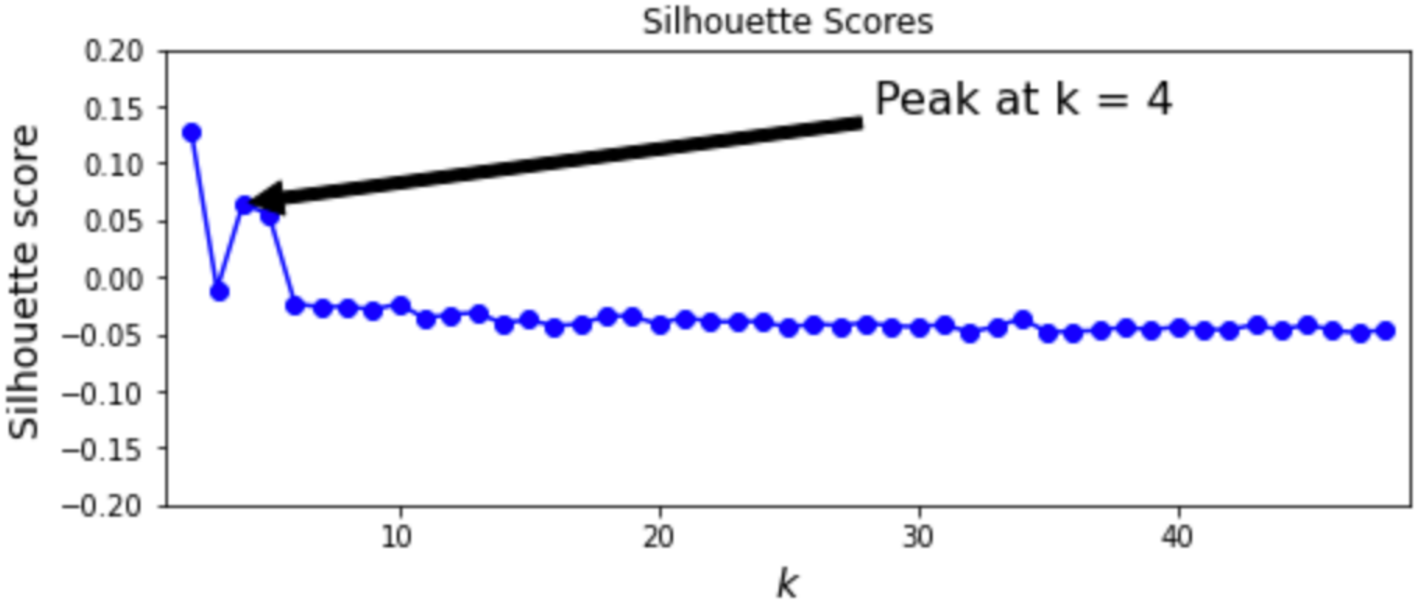

Figure S1B

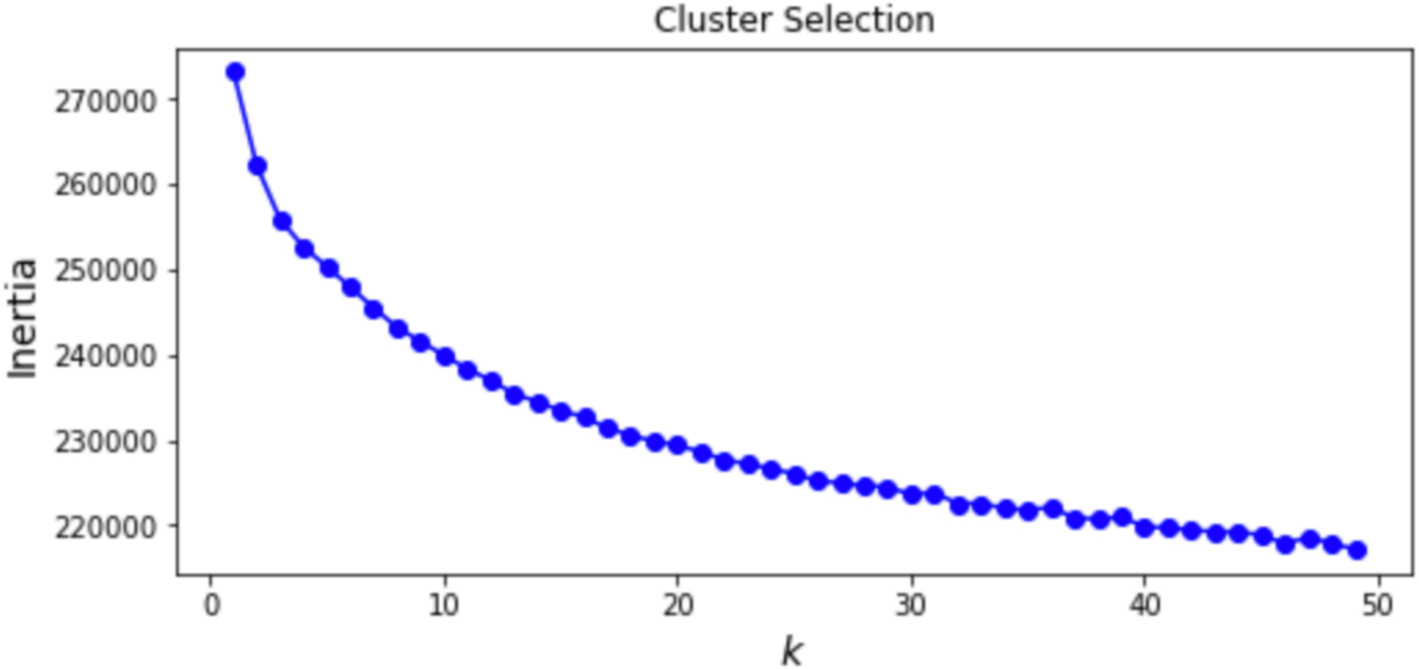

Figure S2A

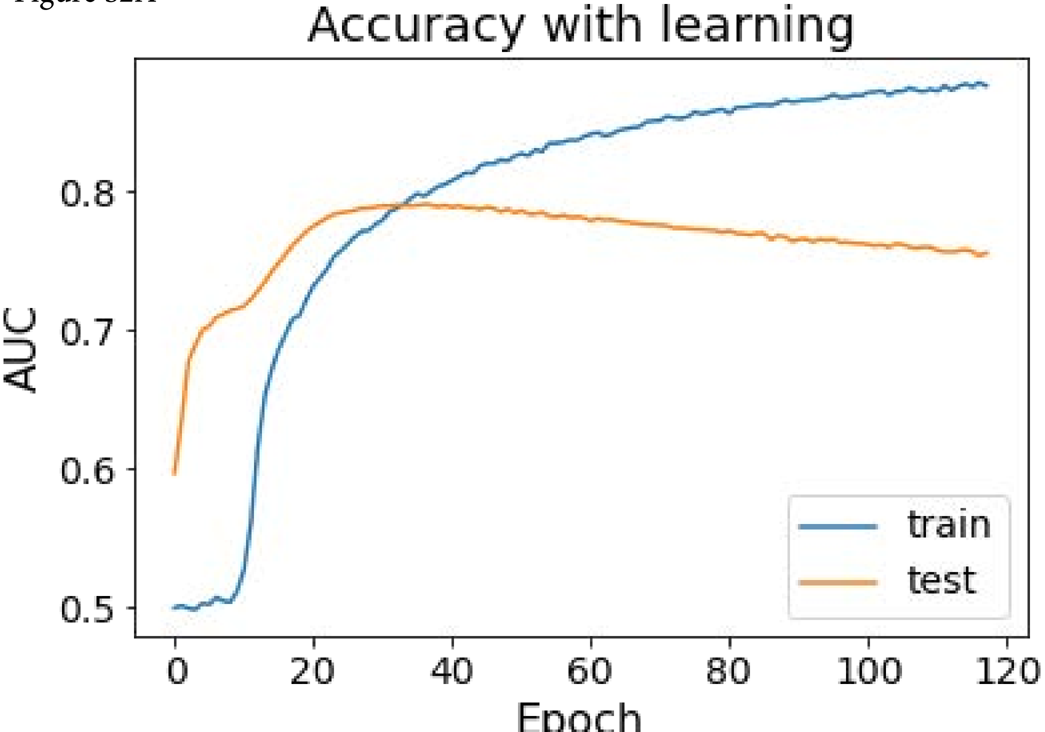
Accuracy with learning
AUC
0.5
0.6
0.7
0.8
0
20
40
60
80
100
120
Epoch
train
test

Figure S2B

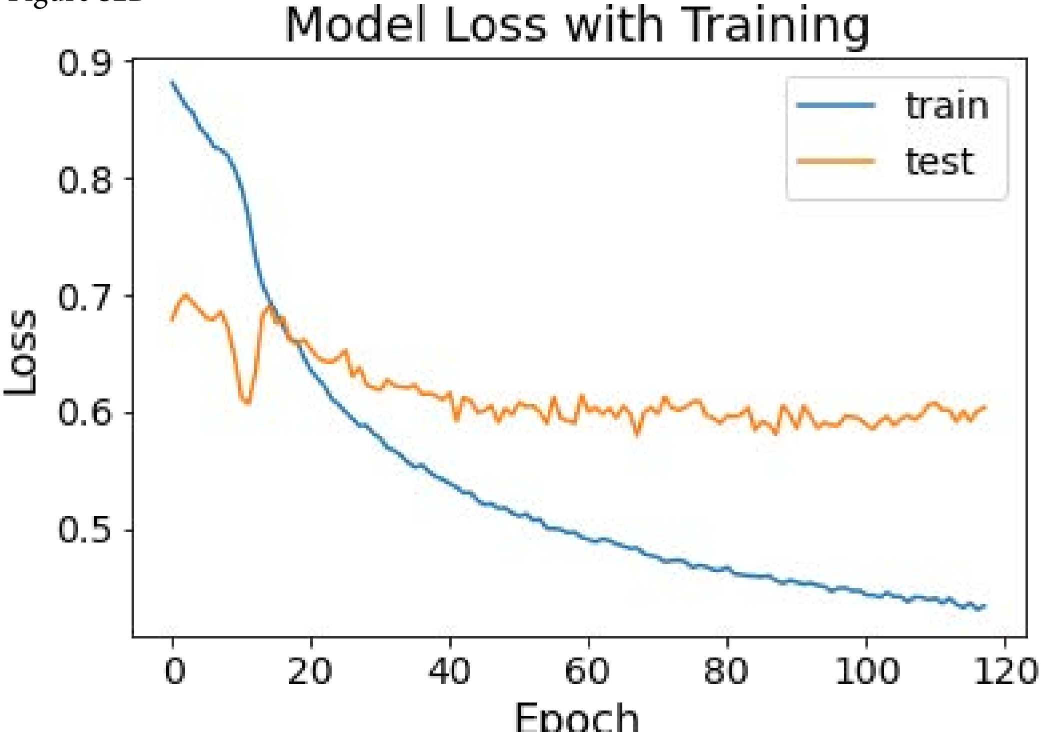

Model Loss with Training
train
test
Loss
Epoch
0.9
0.8
0.7
0.6
0.5
0
20
40
60
80
100
120

Table S1.

Contingency Tables for QT-associated meds (whole population)

Amiodarone (Chi2 = 1010.7, p = 1.69E-217)

		diLQTS		
		No	Yes	Total
Drug administered	No	29853	3858	33711
	Yes	1228	700	1928
	Total	31081	4558	35639

Azithromycin (Chi2 = 0.0085, p = 0.999)

		diLQTS		
		No	Yes	Total
Drug administered	No	28689	4209	32898
	Yes	2392	349	2741
	Total	31081	4558	35639

Chlorpromazine (Chi2 = 0.6111, p = 0.962)

		diLQTS		
		No	Yes	Total
Drug administered	No	30917	4538	35455
	Yes	164	20	184
	Total	31081	4558	35639

Cilostazol (Chi2 = 0.0024, p = 0.9999)

		diLQTS		
		No	Yes	Total
Drug administered	No	31053	4554	35607
	Yes	28	4	32
	Total	31081	4558	35639

Ciprofloxacin (Chi2 = 1.983, p = 0.739)

		diLQTS		
		No	Yes	Total
Drug administered	No	30374	4439	34813
	Yes	707	119	826
	Total	31081	4558	35639

Citalopram (Chi2 = 4.455, p = 0.348)

		diLQTS		
		No	Yes	Total
Drug administered	No	30074	4383	34457
	Yes	1007	175	1182
	Total	31081	4558	35639

Clarithromycin (Chi2 = 1.441, p = 0.837)

		diLQTS		
		No	Yes	Total
Drug administered	No	31025	4546	35571
	Yes	56	12	68
	Total	31081	4558	35639

Disopyramide (Chi2 = 1.563, p = 0.815)

		diLQTS		
		No	Yes	Total
Drug administered	No	31076	4556	35632
	Yes	5	2	7
	Total	31081	4558	35639

Dofetilide (Chi2 = 354.8, p = 1.609E-75)

		diLQTS		
		No	Yes	Total
Drug administered	No	30873	4388	35261
	Yes	208	170	378
	Total	31081	4558	35639

Donepezil (Chi2 = 2.943, p = 0.567)

		diLQTS		
		No	Yes	Total
Drug administered	No	30789	4503	35292
	Yes	292	55	347
	Total	31081	4558	35639

Dronedarone (Chi2 = 5.338, p = 0.254)

		diLQTS		
		No	Yes	Total
Drug administered	No	31033	4544	35577
	Yes	48	14	62
	Total	31081	4558	35639

Droperidol (Chi2 = 10.156, p = 0.038)

		diLQTS		
		No	Yes	Total
Drug administered	No	30998	4557	35555
	Yes	83	1	84
	Total	31081	4558	35639

Erythromycin (Chi2 = 2.802, p = 0.591)

		diLQTS		
		No	Yes	Total
Drug administered	No	30480	4453	34933
	Yes	601	105	706
	Total	31081	4558	35639

Escitalopram (Chi2 = 1.973, p = 0.741)

		diLQTS		
		No	Yes	Total
Drug administered	No	30310	4429	34739
	Yes	771	129	900
	Total	31081	4558	35639

Flecainide (Chi2 = 5.877, p = 0.209)

		diLQTS		
		No	Yes	Total
Drug administered	No	30950	4527	35477
	Yes	131	31	162
	Total	31081	4558	35639

Fluconazole (Chi2 = 22.254, p = 0.000178)

		diLQTS		
		No	Yes	Total
Drug administered	No	29766	4295	34061
	Yes	1315	263	1578
	Total	31081	4558	35639

Haloperidol (Chi2 = 3.539, p = 0.472)

		diLQTS		
		No	Yes	Total
Drug administered	No	27663	4014	31677
	Yes	3418	544	3962
	Total	31081	4558	35639

Ibutilide (Chi2 = 6.258, p = 0.181)

		diLQTS		
		No	Yes	Total
Drug administered	No	31061	4550	35611
	Yes	20	8	28
	Total	31081	4558	35639

Levofloxacin (Chi2 = 6.531, p = 0.163)

		diLQTS		
		No	Yes	Total
Drug administered	No	28602	4144	32746
	Yes	2479	414	2893
	Total	31081	4558	35639

Hydroxychloroquine (Chi2 = 2.999, p = 0.558)

		diLQTS		
		No	Yes	Total
Drug administered	No	30855	4514	35369
	Yes	226	44	270
	Total	31081	4558	35639

Methadone (Chi2 = 7.445, p = 0.114)

		diLQTS		
		No	Yes	Total
Drug administered	No	30672	4475	35147
	Yes	409	83	492
	Total	31081	4558	35639

Moxifloxacin (Chi2 = 1.509, p = 0.825)

		diLQTS		
		No	Yes	Total
Drug administered	No	30866	4519	35385
	Yes	215	39	254
	Total	31081	4558	35639

Ondansetron (Chi2 = 188.49, p = 1.121E-39)

		diLQTS		
		No	Yes	Total
Drug administered	No	10797	2060	12857
	Yes	20284	2498	22782
	Total	31081	4558	35639

Oxaliplatin (Chi2 = 1.027, p = 0.906)

		diLQTS		
		No	Yes	Total
Drug administered	No	31074	4558	35632
	Yes	7	0	7
	Total	31081	4558	35639

Papaverine (Chi2 = 1.320, p = 0.858)

		diLQTS		
		No	Yes	Total
Drug administered	No	31072	4558	35630
	Yes	9	0	9
	Total	31081	4558	35639

Pentamidine (Chi2 = 16.072, p = 0.00292)

		diLQTS		
		No	Yes	Total
Drug administered	No	31020	4535	35555
	Yes	61	23	84
	Total	31081	4558	35639

Procainamide (Chi2 = 11.352, p = 0.0229)

		diLQTS		
		No	Yes	Total
Drug administered	No	31073	4552	35625
	Yes	8	6	14
	Total	31081	4558	35639

Propofol (Chi2 = 541.36, p = 7.583E-116)

		diLQTS		
		No	Yes	Total
Drug administered	No	28786	3747	32533
	Yes	2295	811	3106
	Total	31081	4558	35639

Sotalol (Chi2 = 85.038, p = 1.489E-17)

		diLQTS		
		No	Yes	Total
Drug administered	No	30841	4458	35299
	Yes	240	100	340
	Total	31081	4558	35639

Table S2. Maximal information coefficient with diLQTS. Top 100 of 500

Diagnosis prior to medication administration	MIC
Long QT syndrome	0.03142634
potassium chloride	0.02238573
furosemide	0.01908279
amiodarone hcl	0.01508642
magnesium sulfate in water	0.01399784
high_risk_med_total	0.01180625
albumin human	0.01147678
iso-osm	0.0113692
Acidosis	0.01080828
porcine/d5w	0.01053103
Cardiogenic shock	0.01038338
Acute respiratory failure with hypoxia	0.00998423
Unspecified atrial fibrillation	0.00980185
sodium bicarbonate	0.00954799
42731:Atrial fibrillation:Atrial fibrillation	0.00933589
Hypokalemia	0.00921588
metoprolol tartrate	0.00908352
propofol	0.00901566
Paroxysmal atrial fibrillation	0.00879152
heparin sodium	0.00862944
insulin lispro	0.00859705
Acute kidney failure, unspecified	0.00855961
mupirocin	0.00855878
aspirin	0.0084282
dextrose 5 % in water	0.00838126
midazolam hcl/pf	0.00835559
dexmedetomidine in 0.9 % nacl	0.00825711
calcium chloride	0.0081312
vancomycin hcl	0.00778704
Cardiomyopathy, unspecified	0.00769213
51881:Acute respiratry failure:Acute respiratory failure	0.00731302
4280:CHF NOS:Congestive heart failure, unspecified	0.00722159
dobutamine hcl in dextrose 5 %	0.00720788
chlorhexidine gluconate	0.00706415
atorvastatin calcium	0.0070005
Acute systolic (congestive) heart failure	0.00694103
79431:Abnorm electrocardiogram:Nonspecific abnormal electrocardiogram [ECG] [EKG]	0.00686085
Heart failure, unspecified	0.0066589
Acute on chronic systolic (congestive) heart failure	0.00642116
warfarin sodium	0.00626935
fentanyl citrate/pf	0.00620797

sod phosphate	0.00612775
monobasic-dibas	0.00612775
vasopressin	0.00605831
Acute posthemorrhagic anemia	0.00599988
Coagulation defect, unspecified	0.00599796
Thrombocytopenia, unspecified	0.00595823
Hypertensive heart disease with heart failure	0.00591227
Atherosclerotic heart disease of native coronary artery without angina pectoris	0.00584266
Fluid overload, unspecified	0.00583724
Persistent atrial fibrillation	0.00577981
hydralazine hcl	0.00575515
Long term (current) use of anticoagulants	0.00565587
Severe sepsis with septic shock	0.00550569
rocuronium bromide	0.0054924
Encounter for palliative care	0.00547401
melatonin	0.00546392
Cardiac arrest, cause unspecified	0.00546334
Non-ST elevation (NSTEMI) myocardial infarction	0.00524574
spironolactone	0.00518127
cefepime hcl	0.00508298
Ventricular tachycardia	0.00504398
pantoprazole sodium	0.00502998
perflutren lipid microspheres	0.00498332
78551:Cardiogenic shock:Cardiogenic shock	0.00490484
dofetilide	0.00485276
4275:Cardiac arrest:Cardiac arrest	0.00482966
Abnormal electrocardiogram [ECG] [EKG]	0.00482608
Pleural effusion, not elsewhere classified	0.00474127
lorazepam	0.00467004
Acute kidney failure with tubular necrosis	0.00466999
5849:Acute kidney failure NOS:Acute kidney failure, unspecified	0.00449977
Chronic systolic (congestive) heart failure	0.0044823
nicardipine in nacl	0.00446695
iso-osm	0.00442873
etomidate	0.00438684
Acute and subacute hepatic failure without coma	0.00436369
phytonadione (vit k1)	0.00429792
sod phos di	0.00424461
mono/k phos mono	0.00424461
mineral oil/petrolatum	0.00424228
Long term (current) use of aspirin	0.00419492
Shock, unspecified	0.0041697
Nonrheumatic mitral (valve) insufficiency	0.00391764
0.9 % sodium chloride	0.00390696
epinephrine	0.00389227

norepinephrine bit/0.9 % nacl	0.00377945
Unspecified atrial flutter	0.00376687
42821:Ac systolic hrt failure:Acute systolic heart failure	0.00376025
Sepsis, unspecified organism	0.00374831
ondansetron hcl/pf	0.00371976
Hyperosmolality and hypernatremia	0.00371383
Hyperglycemia, unspecified	0.0037078
piperacillin sodium/tazobactam	0.003688
cisatracurium besylate	0.00368624
Hypomagnesemia	0.00367936
4254:Prim cardiomyopathy NEC:Other primary cardiomyopathies	0.00365799

Table S3.

Cluster 0

**6**	Acute kidney failure, unspecified	0	0.599074	0.431350	0.258411
**4**	Sepsis, unspecified organism	0	0.507407	0.463010	0.234935
**7**	Acute respiratory failure with hypoxia	0	0.504630	0.413016	0.208420
**10**	Acidosis	0	0.462037	0.397907	0.183848
**8**	51881:Acute respiratry failure:Acute respirato...	0	0.414815	0.369008	0.153070
**21**	Anemia, unspecified	0	0.445370	0.272714	0.121459
**12**	5849:Acute kidney failure NOS:Acute kidney fai...	0	0.384259	0.313082	0.120305
**14**	Encounter for palliative care	0	0.323148	0.295507	0.095493
**20**	Do not resuscitate	0	0.354630	0.246807	0.087525
**15**	0389:Septicemia NOS:Unspecified septicemia	0	0.302778	0.280931	0.085060
**27**	Pneumonia, unspecified organism	0	0.307407	0.239049	0.073486
**18**	Person on outside of heavy transport vehicle i...	0	0.257407	0.233715	0.060160
**41**	Hypo-osmolality and hyponatremia	0	0.284259	0.194055	0.055162
**22**	99591:Sepsis:Sepsis	0	0.242593	0.220042	0.053380
**29**	Pleural effusion, not elsewhere classified	0	0.259259	0.198653	0.051503
**17**	Severe sepsis with septic shock	0	0.227778	0.221435	0.050438
**37**	2859:Anemia NOS:Anemia, unspecified	0	0.259259	0.188082	0.048762
**30**	Encephalopathy, unspecified	0	0.233333	0.193869	0.045236
**68**	Hypokalemia	0	0.331481	0.136022	0.045089
**25**	Other specified counseling	0	0.207407	0.187664	0.038923

Cluster 1

Diag_med	cluster	max	diff	calc_diff	
1	Atherosclerotic heart disease of native corona...	1	0.760395	0.601135	0.457100
3	Hyperlipidemia, unspecified	1	0.706836	0.436465	0.308509
5	Long term (current) use of aspirin	1	0.595490	0.365860	0.217866
11	Old myocardial infarction	1	0.327696	0.265659	0.087055
9	Presence of coronary angioplasty implant and g...	1	0.311487	0.274450	0.085488
13	41401:Crnry athrscl natve vssl:Coronary athero...	1	0.269204	0.233093	0.062749
50	Family history of ischemic heart disease and o...	1	0.317125	0.192125	0.060928
16	41400:Cor ath unsp vsl ntv/gft:Coronary athero...	1	0.251586	0.216400	0.054443
53	4019:Hypertension NOS:Unspecified essential hy...	1	0.412262	0.109484	0.045136
19	Presence of aortocoronary bypass graft	1	0.207893	0.180115	0.037445
76	Personal history of nicotine dependence	1	0.431290	0.083141	0.035858
87	Chest pain, unspecified	1	0.229035	0.138327	0.031682
58	Type 2 diabetes mellitus without complications	1	0.319239	0.097943	0.031267
89	78650:Chest pain NOS:Chest pain, unspecified	1	0.190275	0.121967	0.023207
95	Gastro-esophageal reflux disease without esoph...	1	0.392530	0.053641	0.021056
31	Non-ST elevation (NSTEMI) myocardial infarction	1	0.186751	0.108048	0.020178
59	Obstructive sleep apnea (adult) (pediatric)	1	0.239605	0.074791	0.017920
40	41070:Subendo infarct, unspec:Subendocardial i...	1	0.138125	0.108496	0.014986

Diag_med	cluster	max	diff	calc_diff	
**63**	Long term (current) use of insulin	1	0.214940	0.066792	0.014356
**55**	Long term (current) use of anticoagulants	1	0.257928	0.049595	0.012792

Cluster 2

Diag_med	cluster	max	diff	calc_diff	
**373**	Nausea	2	0.073838	0.018557	0.001370
**303**	Single live birth	2	0.015487	0.015487	0.000240
**327**	Other specified diseases and conditions compli...	2	0.015487	0.013512	0.000209
**349**	Unspecified motorcycle rider injured in collis...	2	0.013274	0.011422	0.000152
**441**	Alcohol dependence with withdrawal, uncomplicated	2	0.012998	0.001887	0.000025
**482**	Cocaine abuse, uncomplicated	2	0.011615	0.001430	0.000017
**487**	Alcohol dependence with intoxication, unspecified	2	0.005808	0.002104	0.000012
**489**	Intervertebral disc disorders with radiculopat...	2	0.006637	0.000295	0.000002

Cluster 3

Nausea with vomiting, unspecified	3	1.000000	0.923148	0.923148	
**2**	78701:Nausea with vomiting:Nausea with vomiting	3	0.583416	0.550082	0.320927
**106**	Unspecified abdominal pain	3	0.197433	0.125807	0.024839
**119**	Epigastric pain	3	0.141165	0.088344	0.012471
**125**	78900:Abdmnal pain unspcf site:Abdominal pain,...	3	0.131293	0.086492	0.011356

**165**	78906:Abdmnal pain epigastric:Abdominal pain, ...	3	0.090819	0.053762	0.004883
**258**	Dizziness and giddiness	3	0.078973	0.039980	0.003157
**343**	Headache	3	0.074038	0.021493	0.001591
**294**	7804:Dizziness and giddiness:Dizziness and gid...	3	0.050346	0.028222	0.001421
**162**	2768:Hypopotassemia:Hypopotassemia	3	0.112537	0.004204	0.000473
**391**	7840:Headache:Headache	3	0.034551	0.009938	0.000343
**439**	78909:Abdmnal pain oth spcf st:Abdominal pain,...	3	0.031589	0.009189	0.000290
**479**	57420:Cholelithiasis NOS:Calculus of gallbladd...	3	0.017769	0.005324	0.000095
**287**	Other diseases of stomach and duodenum	3	0.030602	0.001898	0.000058

Tables S4. Contingency Tables

Table S4A. Deep Learning

		Actual		
		0	1	
**Predicted**	0	4424	247	4671
	1	1792	665	2457
		6216	912	7128

Table S4B. Clusters

		Actual		
		0	1	
**Predicted**	0	4250	420	4670
	1	1966	492	2458
		6216	912	7128

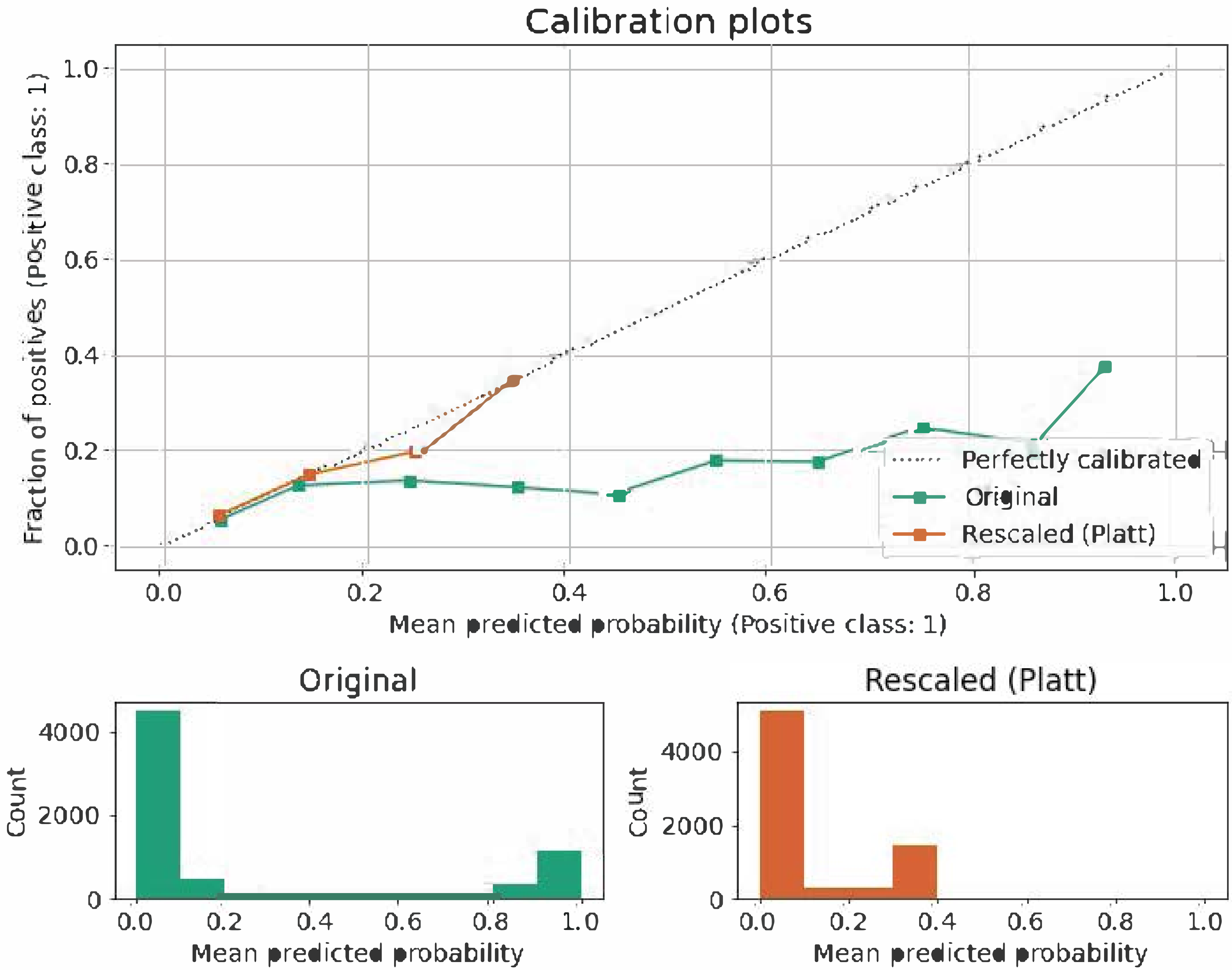

Figure S3A. Calibration plot for neural network with (orange) and without (green) rescaling (Platt method).

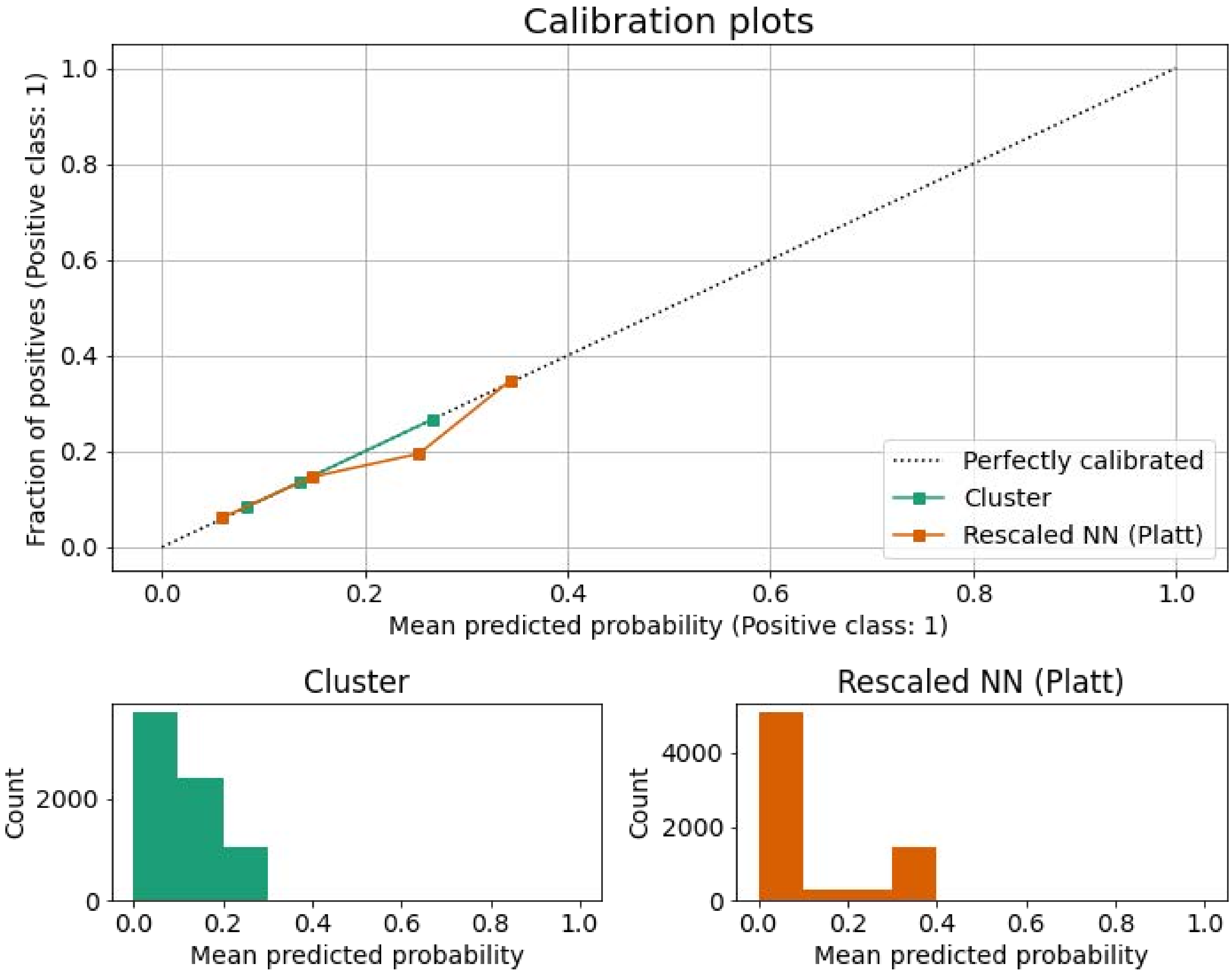

Figure S3B. Calibration Plots comparing cluster prediction with rescaled neural network (Platt rescaling)
